# Conserved Autophagy Pathway Contributes to Stress Tolerance and Virulence and Differentially Controls Autophagic Flux Upon Nutrient Starvation in *Cryptococcus neoformans*

**DOI:** 10.3389/fmicb.2019.02690

**Published:** 2019-11-26

**Authors:** Xueru Zhao, Weijia Feng, Xiangyang Zhu, Chenxi Li, Xiaoyu Ma, Xin Li, Xudong Zhu, Dongsheng Wei

**Affiliations:** ^1^National Key Program of Microbiology and Department of Microbiology, College of Life Sciences, Nankai University, Tianjin, China; ^2^Beijing Key Laboratory of Genetic Engineering Drug and Biotechnology, Institute of Biochemistry and Molecular Biology, School of Life Sciences, Beijing Normal University, Beijing, China

**Keywords:** *Cryptococcus neoformans*, *ATG* genes, autophagy, Atg8, starvation, virulence, stress tolerance

## Abstract

Autophagy is mainly a catabolic process, which is used to cope with nutrient deficiency and various stress conditions. Human environment often imposes various stresses on *Cryptococcus neoformans*, a major fungal pathogen of immunocompromised individuals; therefore, autophagic response of *C. neoformans* to these stresses often determines its survival in the host. However, a systematic study on how autophagy related (*ATG*) genes influence on autophagic flux, virulence, stress response and pathogenicity of *C. neoformans* is lacking. In this study, 22 *ATG*-deficient strains were constructed to investigate their roles in virulence, pathogenesis, stress response, starvation tolerance and autophagic flux in *C. neoformans*. Our results showed that Atg6 and Atg14-03 significantly affect the growth of *C. neoformans* at 37°C and laccase production. Additionally, *atg2*Δ and *atg6*Δ strains were sensitive to oxidative stress caused by hydrogen peroxide. Approximately half of the *atg*Δ strains displayed higher sensitivity to 1.5 M NaCl and remarkably lower virulence in the *Galleria mellonella* model than the wild type. Autophagic flux in *C. neoformans* was dependent on the Atg1-Atg13, Atg5-Atg12-Atg16, and Atg2-Atg18 complexes and Atg11. Cleavage of the green fluorescent protein (GFP) from Atg8 was difficult to detect in these autophagy defective mutants; however, it was detected in the *atg3*Δ, *atg4*Δ, *atg6*Δ and *atg14*Δ strains. Additionally, no homologs of *Saccharomyces cerevisiae ATG10* were detected in *C. neoformans*. Our results indicate that these *ATG* genes contribute differentially to carbon and nitrogen starvation tolerance in *C. neoformans* compared with *S. cerevisiae*. Overall, this study advances our knowledge of the specific roles of *ATG* genes in *C. neoformans*.

## Introduction

Autophagy is mainly a catabolic process, which is induced by nutrient deficiency and various stress conditions (Yang and Klionsky, 2010). *Saccharomyces cerevisiae* exhibits two major forms of autophagy: macroautophagy and microautophagy (Reggiori and Klionsky, 2013). Macroautophagy (hereafter referred to as autophagy) is a fundamental function of eukaryotic cells and is well conserved from *S. cerevisiae* to humans (Nakatogawa et al., 2009). The most important and characteristic morphological feature of autophagy is the *de novo* formation of double-membrane autophagosome, which contains either bulk cytoplasm or selected cargos, depending on the inducing conditions (Inoue and Klionsky, 2010). The formation, expansion, fusion with vacuole and degradation of autophagosome in the vacuole constitute major steps in autophagy that depend on a series of autophagy related (*ATG*) genes.

**FIGURE 1:**
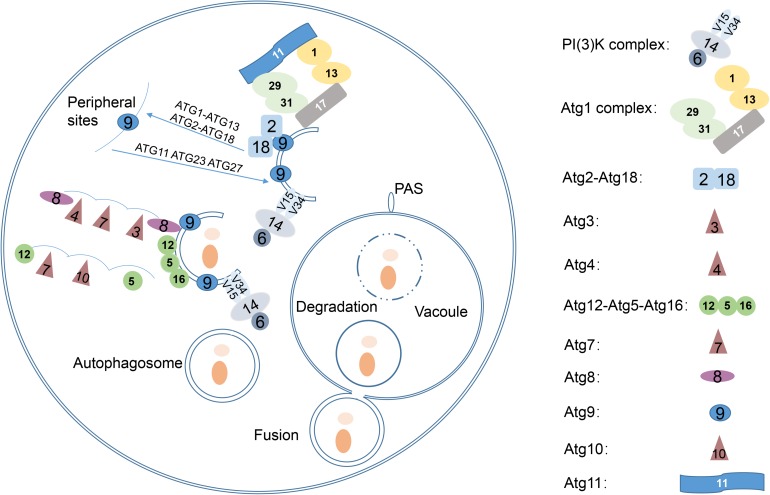
Overview of the autophagy pathway in yeast. Five key stages of autophagy are as follows: (1) Phagophore nucleates at the PAS and sequesters vesicle formation in yeast; (2) phagophore expands to sequester the cargo; (3) phagophore closes the double-membrane autophagosomes; (4) autophagosome fuses with the vacuole, releasing the inner vesicle, known as the autophagic body; (5) autophagic bodies are degraded by vacuolar hydrolases, and the products are released into the cytosol by various permeases. Revised from Nakatogawa et al., 2009.

At present, more than 40 *ATG* genes have been identified in fungi, and the proteins encoded by most of these genes form several complexes during the autophagic process (Lin et al., 2019). *ATG1*, the first identified *ATG* gene, encodes a Ser/Thr kinase (Matsuura et al., 1997). Atg13, which binds to Atg1, is highly phosphorylated by TORC1 (Target of Rapamycin Complex) kinase under nutrient proficient conditions. However, Atg13 is rapidly dephosphorylated upon starvation or rapamycin treatment (Kamada et al., 2000). The Atg1-Atg13 complex comprises the most upstream Atg subfamily in the hierarchy of localization of Atg proteins to the pre-autophagosomal structure (PAS) (Kawamata et al., 2008). Phosphatidylinositol-3-phosphate (PI3P) is essential for autophagosome formation and resides in the autophagosomal membrane. Formation of PI3P depends on the PI3K complex I, which contains Vps34, Vps15, Vps30/Atg6, Atg14 and Atg38 (Petiot et al., 2000; Kihara et al., 2001). Atg8 is a small hydrophilic ubiquitin-like protein, whose accumulation is upregulated upon starvation. Atg8 is immediately cleaved by cysteine protease Atg4 to expose the C-terminal glycine (Gly) residue, which is essential for subsequent conjugation reactions (Kirisako et al., 1999, 2000). In conjugation with phosphatidylethanolamine (PE), Atg8 is involved in the elongation of the isolation membrane; this is mediated by Atg7 and Atg3, which are E1 and E2 enzymes in the ubiquitylation reaction, respectively (Ichimura et al., 2000). Atg4 is also a deconjugation enzyme that liberates Atg8 from the Atg8-PE complex in the outer membrane of autophagosome after fusion with the vacuole (Kirisako et al., 2000). Similar to the Atg8-PE system, Atg12 is catalyzed by Atg7 (E1 enzyme) and Atg10 (E2 enzyme) and then conjugated with Atg5 via an isomerized peptide bond with a lysine residue at the middle of Atg5 (Mizushima et al., 1998). the Atg12-Atg5 complex has an E3-like function in the lipidation of Atg8 (Hanada et al., 2007). Atg16 is required for the localization of Atg12-Atg5 to PAS in a PI3P-dependent manner (Suzuki et al., 2007). Atg9 is the only known integral membrane protein involved in the regulation of autophagosome formation by interacting with the Atg18-Atg2 complex in a PI3P-dependent manner (Noda et al., 2000; Obara et al., 2008) (Figure 1).

*C. neoformans* is a major fungal pathogen of immunocompromised hosts, such as individuals who have human immunodeficiency virus (HIV)/AIDS or are subjects of organ transplant and long-term corticosteroid treatment (Park et al., 2009). *C. neoformans* infection is generally caused by the inhalation of yeast cells or spores that colonize the lung, resulting in cause pulmonary infection, and then disseminate through blood vessels, causing cryptococcal meningitis (Rajasingham et al., 2017). Recent studies have shown that several *ATG* genes are involved in the virulence of *C. neoformans*. Deletion of *VPS34* leads to defective formation of Atg8-labeled vesicles and dramatic attenuation of virulence in mouse models of infection (Hu et al., 2008). Knockdown of the *ATG8* gene led to higher sensitivity to starvation stress and attenuated virulence in both intravenous and intranasal murine models (Hu et al., 2008). Deletion of *ATG7* led to increased cellular size and decreased survival rate in the lungs of infected mice (Oliveira et al., 2016). A recently published study suggests that different *ATG* genes are also involved in non-autophagic functions and contribute to virulence beyond their core functions in autophagy (Ding et al., 2018).

Despite these studies, a systematic investigation of how these *ATG* genes affect stress response, virulent factors, pathogenicity and autophagic flux under carbon and nitrogen starvation conditions is lacking. In this study, we generated 22 *ATG* mutants, and investigated the impacts of these genes on autophagic flux, virulence, stress response and pathogenicity of *C. neoformans*.

## Materials and Methods

### Strains, Plasmids and Culture Conditions

*C. neoformans* var. *grubii* strain H99 was used as the wild type (WT) in this study. The CRISPR-Cas9 system was used to knock out 22 *ATG* genes in *C. neoformans*. All vectors were amplified in *Escherichia coli* DH5α using ampicillin as the selection marker. Hygromycin B-resistance or Geneticin 418-resistance was used as the selection marker for transformed yeast cells. Yeast strains were cultured at 30°C with shaking at 180 rpm. The complete YPD medium (2% glucose, 2% Bacto peptone and 1% yeast extract [pH 6.0]) supplemented with 200 μg/mL hygromycin B or 40 μg/mL G418 were used for routine growth. Nitrogen starvation medium (SD-N; 0.17% yeast nitrogen base [without amino acids and ammonium sulfate] and 2% glucose), carbon starvation medium (SD-C; 0.17% yeast nitrogen base [without amino acids and ammonium sulfate], 0.5% ammonium sulfate, 0.5% casamino acids, 0.002% tryptophan, 0.002% adenine and 0.002% uracil) (Adachi et al., 2017), Nitrogen and carbon starvation medium (PBS; 0.8% NaCl, 0.02% KCl, 0.15% Na_2_HPO_4_ and 0.024% KH_2_PO_4_ [pH 7.4]) were used separately for nitrogen, carbon, nitrogen and carbon starvation treatments, respectively.

### Construction of *ATG* Deletion (*atgΔ*) Mutants and Green Fluorescent Protein (GFP)-Atg8 Expression Strains

Each of the 22 *ATG* genes was deleted using the CRISPR-Cas9 system described previously (Wang et al., 2016). The plasmid contained double *Bsp*QI restriction sites between the U6 promoter and guide RNA. The target sequences (guiding RNA) were inserted into *Bsp*QI sites by annealing two reverse complementary sequences (TTG-N19/AAC-N19), followed by ligation using T4 ligase. Two homologous fragments were ligated to flank the hygromycin resistance (*HygR*) gene by double digestion with *Cla*I/*Hin*dIII and *Bam*HI/*Xba*I, followed by ligation with T4 DNA ligase or In-Fusion HD Cloning kits (Takara). The pBS-Hyg-Atg-Cas9-gDNA plasmid was linearized using either *Kpn*I or *Xba*I, and 1.5 μg of linearized fragments were transformed into the strain H99 by electroporation (Wang et al., 2016).

To construct the EGFP-Atg8 fusion protein expression plasmid, the *ATG8* promoter (1,000 bp upstream of the start codon) and *EGFP* open reading frame (ORF) were ligated by overlapping PCR, and the resultant fragment was inserted into the *Kpn*I site in pBS-G418 by In-Fusion HD Cloning to produce an intermediate plasmid. The ORF and terminator of *ATG8* were then amplified from the genome of *C. neoformans* and ligated into the *Cla*I/*Hind*III sites of the intermediate plasmid to generate final plasmid pBS-G418-EGFP-Atg8. The final plasmid was linearized using *Kpn*I, and 3 μg of the linearized product was transformed into the strain H99 and *atg*Δ mutants by electroporation. Yeast strains used in this study were listed in the Supplementary Table S1. Primers used in this study were listed in the Supplementary Table S2.

### *In vitro* Virulent Factors Assays

Overnight (16-h) cultures in YPD liquid medium were harvested by centrifugation and washed three times with sterile PBS. Ten-fold gradient dilutions of WT and mutant strains were spotted on YPD agar plates and incubated at 37°C to investigate the thermotolerance of mutants. Laccase activity was assayed on Asn agar (0.1% glucose, 100 mg/L norepinephrine [TCI, Tokyo, Japan], 0.05% MgSO_4_-7H_2_O) by incubation in the dark for 3 days. Capsule formation was induced by incubating the WT and mutant cells in maltose medium (2% maltose, 0.1% Bacto peptone and 0.2% yeast extract) at 30°C for 4 days. Cells were stained with India ink and observed under a Nikon Eclipse 80i fluorescence microscope (Nikon, Tokyo, Japan).

To quantify laccase activity, overnight cultures were transferred to 0.1% Asn liquid medium for 5 h and diluted to a concentration of 1 × 10^8^ cells/mL. Then, 100 μL of each yeast culture was placed in a 48-well clear flat-bottom plate. Then, 80 μL of 20 mg/mL 2, 2′-azino-bis (3-ethylbenzothiazoline-6-sulfonic acid) (ABTS) and 820 μL of 0.1 M Sodium acetate (pH 6.5) was added to each culture. Sterile 0.1% Asn liquid medium without any culture was used as a negative control. The plate was incubated at 30°C for 3 h. The absorbance of each culture was measured at 420 nm. The final EU of each strain was calculated using the following equation, modified from Alvarado-Ramírez et al., 2008:

(A⁢ 420180⁢min-A⁢4200⁢min)⁢/⁢107⁢cell/mL=⁢(EU/107⁢cells/mL)

### Stress Response and Drug Sensitivity Assays

Ten-fold gradient dilutions of WT and *atg*Δ mutant cultures were spotted on YPD agar medium containing 0.01% SDS, 1.5 M NaCl, 2 M KCl, 1% Congo red, 10 mM H_2_O_2_, 1 M sorbitol and various antifungal drugs including Itraconazole (0.01 μg/mL), Fluconazole (6 μg/mL), Amphotericin B (0.1 μg/mL) and incubated at 30°C for 3 days.

### Total RNA Isolation and Quantitative Real-Time PCR (qRT-PCR) Analysis

Total RNA was isolated using the RNAprep pure Plant Kit (Tiangen). Approximately 500 ng of total RNA was reverse-transcribed with oligo dT18 primer. Then, qRT-PCR was performed using 10 μL of SYBR green master mix (Roche), 0.5 μL of each primer, 8 μL of DNase/RNase-free water and 1 μL of cDNA or distilled water (negative control). Gene expression levels were determined using the ΔΔC_T_ method, and the *Actin1* (*ACT1*) gene was used for data normalization.

### Pathogenicity of *atg*Δ Mutants in *Galleria mellonella*

The pathogenicity of *atg*Δ mutants in *G. mellonella* was assessed as described previously (Mylonakis et al., 2005). Larvae (0.2–0.3 g) without any dark spots were incubated at 28°C until the day before infection. Yeast cells were grown overnight in YPD liquid medium, washed three times with PBS and resuspended in PBS containing kanamycin (50 μg/mL) to a final concentration of 1 × 10^8^ cells/mL. Then, 20 larvae injected into the last right proleg with 10 μL of yeast suspension. Another group of 20 larvae injected with 10 μL of PBS containing kanamycin served as a control. Larvae were incubated at 28°C, and their survival rate was monitored daily. Survival curves were drawn using the GraphPad Prism 5 software (GraphPad, San Diego, CA, United States).

### Starvation Survival Assays

Yeasts were grown to mid-log phase in YPD liquid medium, harvested, washed three times with sterile PBS, and resuspended in 5 mL of SD-N, SD-C or PBS to a final concentration of 1 × 10^8^ cells/mL. The cultures were incubated at 30°C with shaking at 180 rpm and starved for different durations. Subsequently, serially diluted cell suspensions at a starting concentration of 1 × 10^8^ cells/mL were spotted on YPD agar plates. The plates were incubated at 30°C for 3 days to determine survival under starvation conditions.

### Fluorescence Microscopy and Western Blotting

Yeast cells expressing EGFP-Atg8 were grown to mid-log phase in YPD medium, starved in SD-N or SD-C medium for 4 h and observed by both fluorescence microscopy and differential interference contrast microscopy (DIC) using laser scanning confocal microscope LSM710 (Zeiss, Germany).

Total protein was extracted by resuspending the cell pellet in 100 μL RIPA lysis buffer (1% PMSF, 1% PIC mix) containing an equal volume acid-washed glass bead. The mixture was vortexed for 1 min, incubated on ice for 1 min, and repeated five times. The lysate was obtained by centrifugation at 12,000 rpm for 10 min at 4°C. The concentration of yeast lysate was determined with using the BCA kit. Then, equal amount of total protein was mixed with 6 × SDS loading buffer (300 mM Tris-HCl [pH 6.8], 12% SDS, 60% glycerin, 0.6% bromophenol blue and 0.6% β-mercaptoethanol) and boiled for 5 min. Western blotting was performed as described previously (Zhu et al., 2001). Briefly, samples were separated by 12% SDS-PAGE at a constant voltage of 100 mV for approximately 2 h. Then, the separated yeast proteins were transferred to a PVDF membrane using *Trans* Blot Turbo (BioRad). The membrane was blocked by incubation in 5% (w/v) skim milk at 25°C for 2 h. To detect the GFP-Atg8 and internal control, membrane was incubated with anti-GFP (1:3,000; Santa Cruz Biotechnology) or anti-β-actin (1:3,000; GenScript) primary antibody, followed by anti-mouse IgG HRP-linked secondary antibody (1:5,000; Cell Signal). The ratio of cleaved to non-cleaved GFP protein was measured to monitor the autophagy flux.

## Results

### Characterization of *ATG* Genes in *C. neoformans*

**FIGURE 2:**
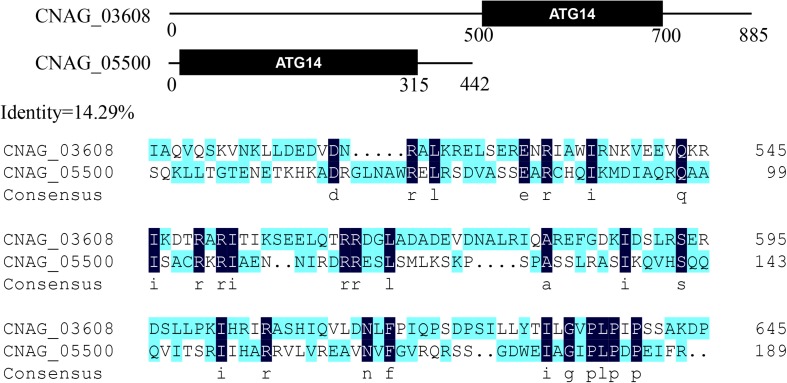
Two Atg14 homologous proteins in *Cryptococcus neoformans*. Putative conserved domains of Atg14-03 (CNAG_03608) and Atg14-05 (CNAG_05500), and amino acid sequence alignments of two Atg14 homologs are shown.

Amino acid sequences of *S. cerevisiae* Atg proteins were used to identify Atg orthologs in *C. neoformans* var. *grubii* H99 strain^1^. A total of 23 Atg proteins were identified, based on the presence of conserved domains (Table 1). However, the genome of *C. neoformans* strain H99 did not contain orthologs of Atg10, an important and specific E2 enzyme required for Atg12-Atg5 conjugation (Shintani et al., 1999). Although Tongbao Liu’s group has claimed the identification of Atg10 in *C. neoformans*, it showed extremely low similarity to known homologs, implying that the role of this protein in Atg12-Atg5 conjugation needs further verification (personal communication). Orthologs of Atg17, 19, 21, 23, 29 and 31 were also not found in *C. neoformans*, possibly because of the very low level of similarity or the absence of these proteins in *C. neoformans*. Interestingly, a homolog of mammalian Atg101 (CNAG_07646) was identified in *C. neoformans*; this represents one of the major difference between *S. cerevisiae* and higher eukaryotes, as Atg29 and Atg31 in S. cerevisiae have been replaced by Atg101 in mammals (Hegedüs et al., 2014; Nanji et al., 2017). This suggests that *C. neoformans* is closer to mammals than to *S. cerevisiae*, which has important implications given that *C. neoformans* is a human pathogen. Two Atg14 homologous proteins (CNAG_03608 and CNAG_05500, later denoted as Atg14-03 and Atg14-05, respectively) were also identified in *C. neoformans*; Atg14 is a specific subunit of one of the PtdIns 3-kinase complexes responsible for targeting the complex to the probable site of autophagosome formation (Figure 2).

**TABLE 1:** Homologs of autophagy-related (ATG) proteins in *C. neoformans*.

**Gene name**	**Broad annotation**	**Function**	**References**
*ATG1*	CNAG_05005	Ser/Thr kinase	Matsuura et al., 1997
*ATG2*	CNAG_06732	Forms a complex with Atg18; involved in Atg9 dynamics at the PAS	Obara et al., 2008
*ATG3*	CNAG_06892	Specific E2 enzyme for Atg8-PE formation	Ichimura et al., 2000
*ATG4*	CNAG_02662	Cysteine protease and deconjugation enzyme; involved in the formation and cleavage of Atg8-PE	Kirisako et al., 2000
*ATG5*	CNAG_06519	Target of Atg12 and interacts with Atg16	Mizushima et al., 1998
*ATG6*	CNAG_01773	Also known as Vps30, a subunit of PI3K	Kihara et al., 2001
*ATG7*	CNAG_04538	Common E1 enzyme for Atg12-Atg5 and Atg8-PE formation	Ichimura et al., 2000
*ATG8*	CNAG_00816	Ubiquitin-like protein conjugated to PE	Kirisako et al., 1999; Kirisako et al., 2000
*ATG9*	CNAG_01445	Integral membrane protein	Noda et al., 2000
*ATG11*	CNAG_01424	Acts as an adapter protein in selective autophagy	Yorimitsu and Klionsky, 2005
*ATG12*	CNAG_07645	Ubiquitin-like protein and conjugated to Atg5	Mizushima et al., 1998
*ATG13*	CNAG_00778	Phosphorylated by TORC1 and regulates Atg1 activity	Kamada et al., 2000
*ATG14-03*	CNAG_03608	Recruits the PI3K complex to the PAS	Itakura et al., 2008
*ATG14-05*	CNAG_05500		
*ATG15*	CNAG_01601	Encodes a putative lipase responsible for the disintegration of autophagic bodies	Teter et al., 2001
*ATG16*	CNAG_02576	Required for the localization of Atg12-Atg5 to PAS	Mizushima et al., 1998
*ATG18*	CNAG_02269	Binds to PI3P and regulates vacuole size	Obara et al., 2008
*ATG20*	CNAG_02730	Sorting nexin family, required for organelle autophagy and contribute to general autophagy	Zhao et al., 2016
*ATG22*	CNAG_07685	Enables the reuse of the resulting macromolecules in the cytosol	Yang et al., 2006
*ATG24*	CNAG_01554	Sorting nexin required for organelle autophagy; contributes to general autophagy	Zhao et al., 2016
*ATG26*	CNAG_12261	Essential for the degradation of very large methanol-induced peroxisomes	Yamashita et al., 2007
*ATG27*	CNAG_04406	Second transmembrane cycling protein; localizes to the Golgi	Yen et al., 2007
*ATG101*	CNAG_07646	Unique subunit of the Atg1-Atg13 complex required in mammals	Hegedüs et al., 2014; Nanji et al., 2017

### Confirmation of the Deletion of the 22 *ATG* Genes

To investigate the function of *ATG* genes, we replaced the genomic copy of each *ATG* gene with the *HygR* gene using the CRISPR-Cas9 suicide cassette system (Supplementary Figure S1A). To confirm whether the replacement cassette was correctly inserted, the putative *atg*Δ transformants were screened by PCR with three primer pairs, Atg-LF/Hyg-R, Hyg-F/Atg-LR, and Atg-LF/Atg-LR, in the last electropherogram, which produced 1,212-bp, 1561-bp and 4,532-bp PCR products, respectively, in the true transformants, whereas the WT produced a 2,426-bp PCR product using the Atg-LF/Atg-LR primer pair (Supplementary Figure S1B). The *atg*Δ strains produced a band that was 2,106 bp larger than H99 because of the insertion of hygromycin cassette. No PCR product could be amplified from the H99 genome using the primer pairs Atg-LF/Hyg-R and Hyg-F/Atg-LR (data not shown).

### Contribution of *ATG* Genes to Autophagic Flux Under Nitrogen and Carbon Starvation Conditions

Studies have shown that *ATG* genes regulate autophagic flux in *S. cerevisiae*; however, whether these genes play similar roles in *C. neoformans* needs further investigation. One method to monitor autophagic flux is to follow the release of free GFP moiety from the GFP-Atg8 fusion protein by western blotting after phagosomes fuse with the vacuole (Cheong and Klionsky, 2008). To confirm that *ATG* genes are functionally associated with autophagy in *C. neoformans*, we constructed 15 strains expressing GFP-Atg8 fusion protein, and analyzed the release of GFP-Atg8 in these mutants.

**FIGURE 3:**
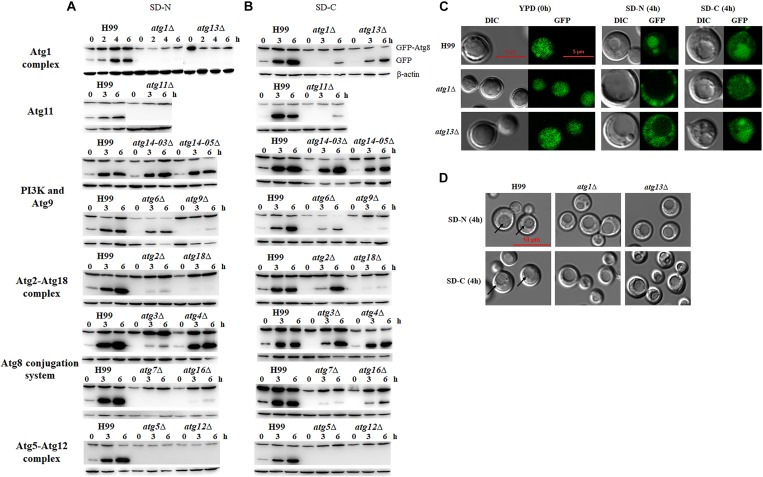
Nitrogen or carbon starvation induces autophagy in *C. neoformans*. **(A,B)** Analysis of GFP-Atg8 processing by western blotting. H99 and *atg*Δ strains transformed with a plasmid expressing the EGFP-Atg8 fusion protein were collected after shifting to nitrogen starvation medium (SD-N) **(A)** or carbon starvation medium (SD-C) **(B)** at the indicated times. Total proteins were extracted and subjected to immunoblot analysis; β-actin was used as the loading control. **(C)** Localization of EGFP-Atg8 upon nutrient starvation. H99, *atg1*Δ and *atg13*Δ cells in log phase (starved; 0 h) or after shifting to SD-N or SD-C medium containing 10 μg/mL nocodazole for 4 h were observed by both fluorescence microscopy and differential interferential contrast (DIC) with a laser scanning confocal microscope. **(D)** Autophagic body formation. H99 and *atg*Δ strains were incubated in SD-N or SD-C medium containing 1 mM PMSF and 10 μg/mL nocodazole for 4 h and subjected to DIC microscopy. Arrows indicate autophagic bodies within vacuoles.

We first analyzed autophagic flux induced under nitrogen starvation conditions. The results showed that the deletion of *ATG1, ATG13, ATG11, ATG7*, *ATG5* and *ATG12* led to significantly lower autophagic flux, as no GFP release was detected in the mutant strains after nitrogen starvation for 6 h, whereas significant GFP release was detected in the WT, indicating that these genes are essential for boosting autophagic flux under nitrogen starvation conditions (Figure 3A). We also found that autophagic flux decreased in *atg2*Δ, *atg9*Δ, *atg18*Δ and *atg16*Δ mutants under nitrogen starvation conditions, indicating that autophagic flux is also dependent on these proteins to some extent. However, deletion of Atg6 and Atg14, two subunits of the PI3K complex I, did not affect the cleavage of GFP-Atg8. Autophagic bodies were easily observed in the *atg6*Δ and *atg14*Δ mutants under nitrogen starvation, indicating the *ATG6* and *ATG14* are not essential for the formation of autophagic bodies (Supplementary Figure S2). Deletion of two components of Atg8 ubiquitin-like conjugation system, Atg3 and Atg4, also did not affect the cleavage of GFP-Atg8. These results suggest that these genes are not directly be involved in the regulation of autophagic flux in *C. neoformans*.

Carbon is essential for all living organisms, and the energy produced by the metabolism of carbon sources can support almost all vital activities. Here, we tested whether deletion of the *ATG* genes affects autophagic flux under carbon starvation conditions, i.e., in the SD-C medium. When the WT cells were transferred from YPD to SD-C medium 3h, the release of GFP from the GFP-Atg8 fusion protein was significantly increased, indicating the boosting effect of glucose starvation on autophagic flux. In mutant cells, only the deletion of *ATG5* and *ATG12* led to the total loss of autophagic flux after starvation for 6 h; however, the deletion of *ATG1*, *ATG7*, *ATG9*, *ATG11*, *ATG16* and *ATG18* resulted in significantly reduced but not total loss of GFP release, indicating their involvement in maintaining autophagic flux upon carbon starvation (Figure 3B).

In comparison with nitrogen starvation, carbon starvation was more efficient in the induction of autophagic flux in *C. neoformans*, as the release of GFP was still visible even after the *ATG* genes were deleted (Figures 3A,B). In other words, these genes play less important roles in controlling autophagic flux under glucose starvation conditions than under nitrogen starvation conditions. Although the deletion of *ATG1* and *ATG11* led to decreased autophagic flux after starvation for 3 h, autophagic flux was enhanced again when mutant cells were incubated for 6 h under glucose starvation conditions. Similar results were obtained for other *ATG* mutants, except *atg5Δ* and *atg12*Δ, indicating that long-term starvation-induced GFP release might not depend on these genes.

To further confirm the roles of these *ATG* genes in the regulation of autophagic flux, localization of GFP fluorescence and formation of autophagic body in the vacuole were observed in the *atg1*Δ and *atg13*Δ mutant cells grown in SD-N or SD-C medium for 4 h by laser scanning confocal microscopy. Fluorescence was detected in the entire cells of the WT and *atg1*Δ and *atg13*Δ mutants under nutrient proficient conditions (Figure 3C). When the cells were incubated in the SD-N medium for 4 h, fluorescence relocated to vacuole in the WT but remained in the cytosol in the *atg1*Δ and *atg13*Δ mutants, confirming the important roles of *ATG1* and *ATG13* in regulating autophagic flux under nitrogen starvation conditions. However, when the cells were shifted from YPD to SD-C for 4 h, partial fluorescence in cytosol of the WT and *atg13*Δ mutant relocated to the vacuole; no relocation was detected in the *atg1*Δ mutant. These results indicated that the *ATG* genes perform non-essential roles in regulating autophagic flux under glucose starvation conditions.

To further confirm this result, autophagic body formation in the vacuole was observed using DIC microscopy under different starvation conditions. Autophagic bodies were observed in vacuole of WT cells under nitrogen starvation conditions but not in the vacuoles of *atg1*Δ and *atg13*Δ mutants, confirming the essential roles of *ATG1* and *ATG13* in autophagic flux. Under glucose starvation conditions, autophagic bodies was observed in the WT but not in the *atg1*Δ and *atg13*Δ mutants (Figure 3D). These results indicated that *ATG* genes play different roles in enhancing autophagic flux under nitrogen and carbon starvation conditions.

### Contribution of *ATG* Genes to Traditional Virulent Factors

**FIGURE 4:**
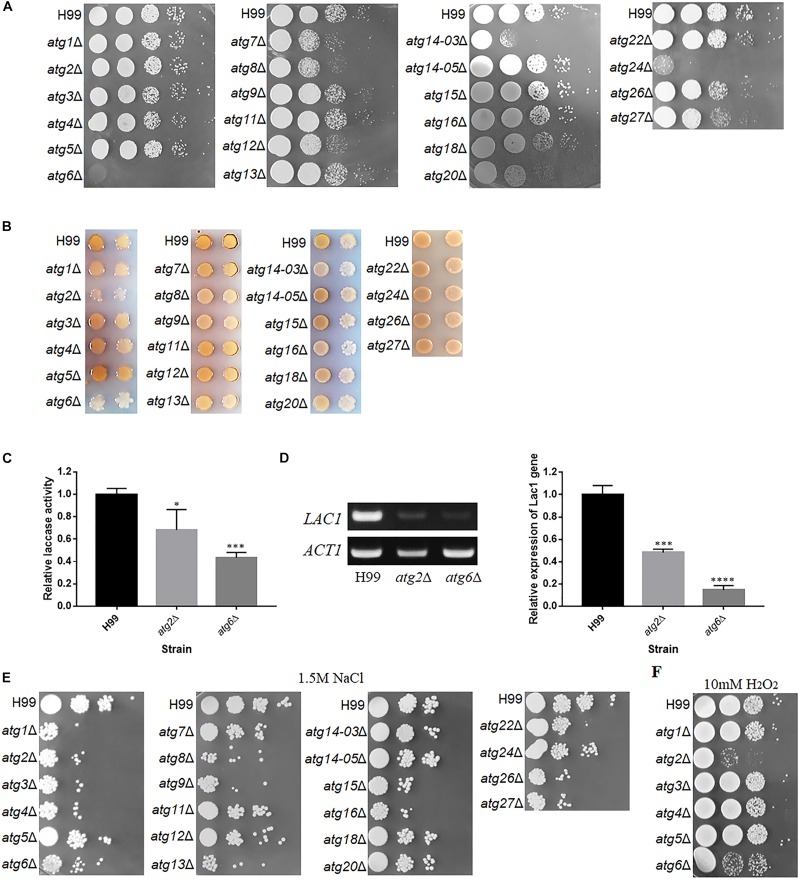
Phenotypes of *atg*Δ mutants of *C. neoformans*. **(A)** Thermotolerance test. Serial 10-fold dilutions of H99 and *atg*Δ strains were spotted onto YPD medium and incubated at 37°C. **(B)** Laccase activity assay to determine melanin production. Each strain was spotted and cultured on Asn medium containing 0.1% glucose and 100 mg/L norepinephrine at 30°C for 3 days. **(C)** Relative laccase activity of H99, *atg2*Δ and *atg6*Δ strains using ABTS as a substrate. The assay was performed three times. Asterisks indicate significant differences between the wild-type (H99) and mutant strains (^∗^*P* < 0.05; ^∗∗∗^*P* < 0.001; Student’s *t*-test). **(D)** Qualitative and quantitative analysis of *LAC1* expression in *atg2*Δ and *atg6*Δ by reverse transcription PCR (RT-PCR) and quantitative real-time (qRT-PCR) upon induction by 0.1% glucose for 5 h. The assay was performed three times. Asterisks indicate significant differences (^∗∗∗^*P* < 0.001; ^****^P < 0.0001; Student’s *t*-test). **(E,F)** Sensitivity of *atg*Δ to NaCl and H_2_O_2_. Serial 10-fold dilutions of H99 and *atg*Δ strains were spotted on agar plates containing 1.5 M NaCl or 10 mM H_2_O_2_ and incubated at 30°C.

*C. neoformans* is a model system for studying fungal pathogenesis and exhibits three well-known pathogenesis-associated virulent factors, including the ability to grow at 37°C, antioxidant melanin and polysaccharide capsule (Williamson, 1997; Zhu and Williamson, 2004). Therefore, we first analyzed these three virulent factors in each of the 22 *atg*Δ mutants. The *atg6*Δ and *atg24*Δ mutants almost completely lost their abilities to grow at 37°C (Figure 4A), indicating that *ATG6* and *ATG24* are essential for thermotolerance. The *atg7*Δ, *atg8*Δ, *atg12*Δ, *atg14-03*Δ and *atg20*Δ mutants also exhibited decreased growth rate at 37°C but continued to propagate slowly, indicating their possible involvement in thermotolerance. Deletion of other *ATG* genes did not affect thermotolerance. Therefore, the contribution of these genes to thermotolerance showed the following order: *ATG6* > *ATG24* > *ATG14-03* > *ATG7*, *8*, *12*, *20* > other *ATG* genes.

The production of melanin was significantly lower in the *atg2*Δ, *atg6*Δ, *atg14-03*Δ and *atg16*Δ mutants compared with the WT (Figure 4B). However, *atg2*Δ and *atg6*Δ mutants showed poor growth on 0.1% Asn, possibly because of inadequate nutrition. Therefore, the impact of the deletion of *ATG2* and *ATG6* on laccase activity was further determined in liquid culture using ABTS as the substrate. The laccase activity of *atg2*Δ and *atg6*Δ decreased by 30 and 60%, respectively, compared with the WT (Figure 4C). Consistent with this observation, the expression of *LAC1*, a laccase gene, was significantly down-regulated to 14.5% in the *atg6*Δ mutant, compared with the WT, as determined by qRT-PCR (Figure 4D). These results confirmed that Atg2 and Atg6 regulate laccase activity and consequently melanin production. However, deletion of other *ATG* genes did not affect the production of melanin.

The size of the capsule induced on maltose medium was also determined for these mutants. We found that the disruption of 20 *ATG* genes led to a smaller capsule than the WT; however, all mutants retained the ability to produce the capsule, implying that these *ATG* genes play insignificant roles in capsule synthesis (Supplementary Figure S3), and most of these genes positively control capsule formation. These results indicate that although *ATG* genes contribute differentially to the three traditional virulent factors, deletion of these genes dramatically affect thermotolerance at physiological temperature.

To confirm these phenotypes, we selected four defective strains (*atg1*Δ,*atg7*Δ,*atg8*Δ, and *atg9*Δ), and its complementation strains were constructed by co-transforming the ATG cassettes and the pBS-G418 plasmid. First, we verified the complementation strains by PCR. WT-sized bands were amplified in the correct transformants, but a larger band was amplified in the defective strains (Supplementary Figure S4A). Then, we measured the high temperature tolerance of these strains as well as capsule induction in low-iron medium and melanin production on L-DOPA agar plates (Ding et al., 2018). The *atg7*Δ and *atg8*Δ strains showed slight sensitivity to high temperatures; however, normal phenotype was restored in the respective complementation strains (Supplementary Figure S4B). The size of capsule in *atg1*Δ, *atg7*Δ, *atg8*Δ, and *atg9*Δ strains was smaller than the WT; this phenotype was also restored in the respective complementation strains (Supplementary Figure S4C). Deletion of none of the four genes affected the production of melanin compared with the WT (Supplementary Figure S4D).

### Contribution of *ATG* Genes to Stress Tolerance and Antifungal Drugs Resistance

In addition to traditional virulent factors, stress tolerance and antifungal resistance of fungal pathogens are also related to pathogenesis. Therefore, WT and *atg*Δ strains were serially diluted and spotted on YPD agar medium containing 1.5 M NaCl, 2 M KCl or H_2_O_2_. Interestingly, *atg1*Δ, *atg2*Δ, *atg3*Δ, *atg4*Δ, *atg6*Δ, *atg8*Δ, *atg9*Δ, *atg13*Δ, *atg15*Δ, *atg16*Δ, *atg26*Δ and *atg27*Δ displayed higher sensitivity to 1.5 M NaCl (Figure 4E) and 2 M KCl (data not shown) than the WT, indicating their contributions to osmotic stress resistance. The *atg2*Δ and *atg6*Δ strains also displayed higher sensitivity to oxidative stress caused by H_2_O_2_ than the WT (Figure 4F), implying these two gene play important antioxidative roles.

The same method was used to determine the sensitivity of these mutants to various antifungal drugs. However, none of the mutants showed high sensitivity to antifungal drugs (Supplementary Figure S5), indicating that either these genes are not involved in antifungal activity to the specific drugs tested in this study or the concentration of drugs used in this investigation was not high enough to elicit a response.

### Contribution of *ATG* Genes to Starvation Tolerance

Because autophagy is important for eukaryotic organisms to withstand starvation tolerance, and survival under starvation condition has been clearly linked to pathogenicity of several pathogens (Hu et al., 2008), starvation tolerance was further analyzed for these *C. neoformans* mutants under nitrogen starvation (SD-N) or carbon starvation (SD-C) or both carbon and nitrogen starvation (PBS) conditions.

**FIGURE 5:**
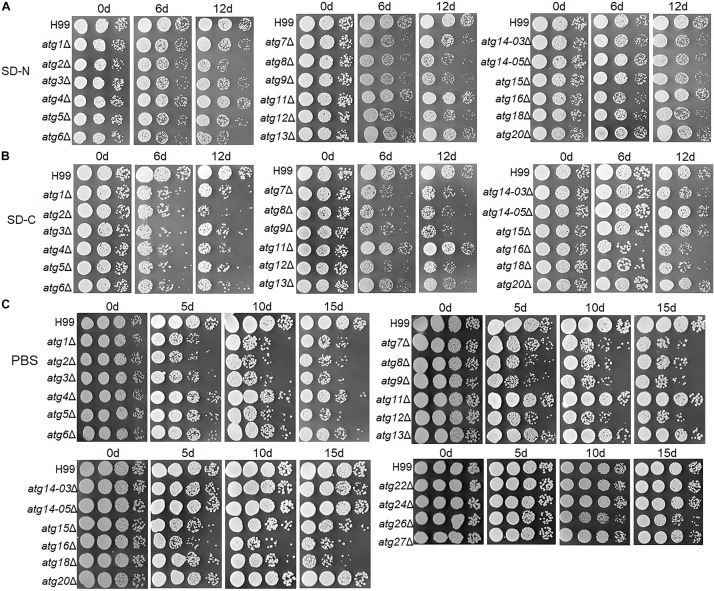
Tolerance of *atg*Δ strains to nitrogen and/or carbon starvation. **(A–C)** H99 and *atg*Δ strains were grown in liquid YPD for 16 h and then grown on SD-N medium **(A)**, SD-C medium **(B)** or PBS **(C)** for the indicated durations. Dilutions were grown on YPD plates for 2 days.

In the 12-day nitrogen starvation treatment, *atg1*Δ, *atg2*Δ, *atg3*Δ, *atg5*Δ, *atg6*Δ, *atg7*Δ, *atg8*Δ, *atg9*Δ, *atg12*Δ, *atg14-03*Δ, *atg14-05*Δ, *atg15*Δ and *atg18*Δ mutants showed lower survival rate than the WT; the survival rate of *atg2*Δ, *atg6*Δ, *atg8*Δ, *atg9*Δ, *atg12*Δ, and *atg18*Δ strains was significantly lower than the WT in SD-N medium (Figure 5A).

In the 6-day glucose starvation treatment, all mutants (except *atg11*Δ, *atg13*Δ, *atg14-03*Δ, *atg14-05*Δ, *atg15*Δ and *atg20*Δ) showed significantly lower survival rate than the WT (Figure 5B).

Under both glucose and nitrogen starvation conditions (i.e., in the PBS buffer), *atg1*Δ, *atg2*Δ, *atg3*Δ, *atg4*Δ, *atg5*Δ, *atg6*Δ, *atg7*Δ, *atg8*Δ, *atg9*Δ, *atg12*Δ, *atg15*Δ, *atg16*Δ and *atg18*Δ mutants showed significantly lower survival rate than the WT. The remaining mutants did not show a drastic reduction in survival rate even after starvation for 15 days (Figure 5C).

These results demonstrate that the *ATG* genes contribute differentially to starvation tolerance, and most these genes play more important roles in tolerance to carbon-only or carbon and nitrogen starvation than in tolerance to nitrogen-only starvation.

### Contribution of *ATG* Genes to the Pathogenicity of *C. neoformans* in the *G. mellonella* Model

**FIGURE 6:**
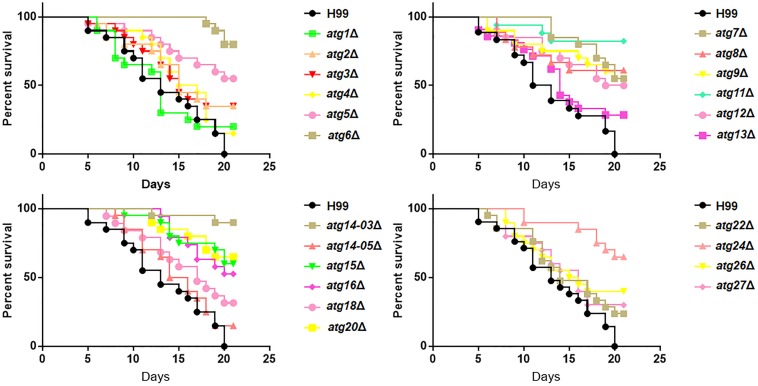
Role of *ATG* genes in the virulence of *C. neoformans*. Survival of *Galleria mellonella* larvae infected with 10^6^ cells of H99 or *atg*Δ strains. The larvae were incubated at 30°C, and their survival was monitored daily.

Our results showed differential roles of *ATG* genes in the regulation of pathogenesis-related virulence. Next, we investigated whether the inference of these virulent factors would eventually lead to a change in pathogenesis. To perform this experiment, we used the *G. mellonella* model, which has been widely used in evaluating pathogenesis of *C. neoformans* (Mylonakis et al., 2005). We found that *atg5*Δ, *atg6*Δ, *atg7*Δ, *atg8*Δ, *atg9*Δ, *atg11*Δ, *atg12*Δ, *atg14-03*Δ, *atg15*Δ, *atg16*Δ, *atg20*Δ and *atg24*Δ strains showed remarkably attenuated pathogenesis in this invertebrate model, whereas *atg6*Δ, *atg11*Δ and *atg14-03*Δ were almost entirely avirulent (Figure 6). The other mutant strains did not show attenuated pathogenesis. Our results further confirmed that PI3K complex I signaling is a virulence-associated trait during infection with *C. neoformans*.

## Discussion

Autophagy is a highly conserved process essential for sustaining cellular integrity, homeostasis and survival (Mizushima et al., 2008). Although approximately 18 core genes have been confirmed to be essential for the critical autophagic steps (Farré and Subramani, 2016), their specific roles need to be investigated in different organisms, considering the influence of evolutionary forces on autophagic processes. In this study, 23 Atg-related homologs of *S. cerevisiae* were identified through BLAST searches of the *C. neoformans* protein database (Table 1), suggesting that the autophagy machinery is relatively well conserved between *S. cerevisiae* and *C. neoformans*.

Interestingly, several *S. cerevisiae ATG* gene homologs were not identified in *C. neoformans* including *ATG10*, *17*, *19*, *21*, *23*, *29* and *31*. The lack of E2-like enzyme Atg10 is an interesting finding, as Atg10 is responsible for catalyzing the conjugation of Atg12 to Atg5 and is essential for autophagosome formation in *S. cerevisiae* (Shintani et al., 1999). However, the appearance of a simple non-covalent system in apicomplexan parasites, *Plasmodium* and *Toxoplasma*, and yeast species, *Komagataella phaffii*, might partially explain the absence of Atg10 in *C. neoformans*. This non-covalent Atg12-Atg5 complex retains the ability to facilitate Atg8-PE conjugation (Pang et al., 2019). Therefore, whether *C. neoformans* also adopts a similar non-covalent interaction system is worth investigating. Atg17 is reportedly conserved among yeast and filamentous fungal species. Although a possible homolog of yeast Atg17 has been found in *C. neoformans* (CNAG_01538) (Palmer et al., 2008), it was actually found to have very low homology to ScAtg17 in the structural maintenance of chromosomes (SMC) domain. Atg19 functions as a receptor for the cytoplasm-to-vacuole targeting (Cvt) cargo and mediate selective autophagy in *S. cerevisiae* (Chang and Huang, 2007). The absence of this homolog in *C. neoformans* indicates that the Cvt process might proceed with a different receptor. Atg21 is also essential for selective autophagy in the Cvt pathway or mitophagy and has been proposed to be required for the recruitment of Atg8 to PAS, especially in the Cvt pathway (Meiling et al., 2004a). Atg23 is required for the maturation proaminopeptidase I but not for autophagy (Meiling et al., 2004b). Thus, our data suggest that either the Cvt pathway is absent in *C. neoformans* or utilizes a different set of enzymes. Atg29 and Atg31 together with Atg17 were found to constitutively form the Atg17-Atg29-Atg31 ternary complex, which acts as an essential scaffold for the organization of PAS in *S. cerevisiae* (Kabeya et al., 2009). The absence of this complex suggests that autophagy induction and PAS organization are completely different in *C. neoformans*. A putative mammalian homolog, Atg101, was unexpectedly found in *C. neoformans*. In mammalian cells, Atg101 is critical for autophagy together with ULK, Atg13 and FIP200 (Jung et al., 2009); however, it is not conserved in budding yeast. Therefore, whether Atg101 in *C. neoformans* plays a similar role as the mammalian Atg101 is an interesting question. Unfortunately, we could not obtain *atg101*Δ mutant cells for unknown reasons.

Only a few *ATG* genes have been reported to be involved in regulating classical virulent factors. Deletion of *Vps34* leads to significantly reduced melanin production (Hu et al., 2008). In our study, only putative *ATG* genes were analyzed. However, because Vps34, Atg14 and Atg6 form a complex to mediate the formation of autophagosome, deletion of *ATG6* and *ATG14* should provide some clues about the function of the PI3K complex. Our results showed that the deletion of *ATG6* and *ATG14-03* affected laccase production, indicating that the PI3K complex does regulate melanin production in *C. neoformans* (Figure 4B). However, deletion of another homolog, Atg14-05, did not result in significantly reduced melanin production, which raises a question regarding the role of Atg14-05 in autophagy in *C. neoformans.* Interestingly, in a previous study, the *atg1*Δ, *atg8*Δ, and *atg9*Δ mutants are not different from the WT in the growth at 30°C and 37°C or in the production of melanin. The *atg7*Δ mutant cause slight growth defect at both 30°C and 37°C and produce less melanin than the WT strain after 48-h incubation. These *atg*Δ mutants are able to produce a polysaccharide capsule similar in size to WT (Ding et al., 2018). However, there are some differences between our study and that of Ding et al. The *atg7*Δ and *atg8*Δ mutants resulted in slight sensitivity to high temperatures, and these four *atg*Δ mutants led to smaller capsules (Figure 4 and Supplementary Figure S4). As studies have pointed out that heat stress induces autophagy to insure protein homeostasis at high temperatures (Zhao et al., 2009), deletion of the heat shock transcription factor 1 (HSF1) increases basal autophagy levels (Dokladny et al., 2013), moreover, the transcriptions of *ATG* genes in *C. neoformans* are significant increase when transferred from 30 to 37°C in YPD medium (Gontijo et al., 2017), we speculate that autophagy can also be induced by high temperature in *C. neoformans*. Atg7 and Atg8 are the core proteins in autophagy process, and they caused slight growth defects at high temperatures which was more significant at 38°C (data not show). Capsules are induced with maltose medium (low nitrogen) and LIM medium (low iron). An important way for responding to low nitrogen and low iron conditions is the Pka1-cyclicAMP (cAMP) pathway (O’Meara and Alspaugh, 2012). Autophagic bodies was also observed in the vacuoles of the WT induced in maltose medium (data not show). However, we cannot explain how the deletion of the *ATG* gene reduces the size of the capsule based on our existing results. Except for the studies on the abovementioned *ATG* genes, no reports are available on the influence of *ATG* genes on virulent factors.

Atg20 and Atg24, the sorting nexins required for selective autophagy including the Cvt pathway, pexophagy and mitophagy (Zhao et al., 2016), were also required for the growth of *C. neoformans* at 37°C, indicating that selective autophagy might also be involved in regulating this virulent factor. A number of *ATG* transcripts have been reported to be induced during macrophage internalization. Suppression of *ATG*8 transcripts with RNAi resulted in severely attenuated fungal virulence, and reduction in the virulence of the *vps34*Δ mutant is caused by a defect in starvation tolerance and loss of the ability to form autophagic bodies during starvation and after phagocytosis by macrophages (Hu et al., 2008). By contrast, autophagy is dispensable for virulence in other pathogenic fungi including *Candida albicans* and *Aspergillus fumigatus* (Palmer et al., 2007; Richie et al., 2007). In our study, 12 *atg*Δ strains showed remarkably attenuated virulence in the invertebrate model of cryptococcosis (Figure 6), demonstrating a close relationship between autophagy and microbial virulence in *C. neoformans*.

Previous research has shown that the high osmolality glycerol (HOG) pathway controls the resistance of cells to osmotic stress in *C. neoformans* (Bahn et al., 2005). As most of *atg*Δ mutants showed reduced growth under high osmotic pressure, whether these *ATG* genes interact with HOG1 (MAPK) pathway to cope with high salt conditions deserves our attention. The *atg2*Δ and *atg6*Δ mutants were also sensitive to 10 mM H_2_O_2_ treatment, which often leads to the production of reactive oxygen species (ROS; Figure 4F). The deletion of *ATG1* in *Candida glabrata* results in higher intracellular ROS than the WT, especially upon induction by H_2_O_2_ (Shimamura et al., 2019). Ethanol stress can also induce autophagy, which is regulated by H_2_O_2_ and superoxide anion (O_2_^–^) derived from the mitochondrial electron transport chain (mtETC), and autophagy in turn contributes to the elimination of H_2_O_2_ and O_2_^–^ (Jing et al., 2018). In *C. neoformans*, whether this relationship exists between ROS elimination and autophagy is worth exploring.

Autophagy is a conserved cellular mechanism essential for survival upon starvation. In this study, most *atg*Δ mutants were sensitive to nitrogen starvation or carbon starvation, especially in the absence of glucose (Figures 5A,B). Under nitrogen starvation conditions, *atg1*Δ, *atg13*Δ, *atg11*Δ, *atg7*Δ, *atg5*Δ *and atg12*Δ mutants completely lost the ability to process GFP-Atg8 (Figure 3A) indicating significantly reduced autophagic flux. The Atg1-Atg13 complex acts as a scaffold for the recruitment of other Atg proteins essential for autophagy, and deletion of ATG1 and ATG13 blocks autophagic flux in S. cerevisiae and S. pombe (Kawamata et al., 2008; Mukaiyama et al., 2009). Atg11 acts as a scaffold for recruiting core Atg proteins and target proteins for selective degradation (Yorimitsu and Klionsky, 2005). Our analyses revealed that Atg11 was necessary for bulk autophagy. Atg7 is a unique E1 enzyme that activates two ubiquitin-like molecules, Atg8 and Atg12, and transfers them to different E2 enzymes (Yamaguchi et al., 2012). The formation of the Atg8-PE complex depends on the activity of the Atg12-Atg5 complex, which functions as an E3-like enzyme to enhance Atg8 lipidation (Hanada et al., 2007). The importance of Atg7, Atg5 and Atg12 in the formation of the Atg8-PE complex is also reflected in our results, and the activity of the E3-like enzyme is not completely dependent on Atg16, which is dispensable for the E3-like function of Atg12-Atg5 *in vitro* but it is required for Atg8-PE formation *in vivo* (Suzuki et al., 2001). The *atg2*Δ, *atg18*Δ and *atg9*Δ mutants only processed a small proportion of GFP-Atg8 in this study. Both these proteins are involved in the formation of autophagosomal membranes (Noda et al., 2000; Obara et al., 2008). Unlike previous studies, residual autophagy activity was detected in the deletion mutants of *ATG6* and *ATG14*, both of which encode subunits of the PI3K complex (Figure 3A and Supplementary Figure S2). Considering that Atg14 has two homologous proteins in *C. neoformans*, single knock does not affect autophagy, which is worthy of further study whether it is due to functional redundancy. In the absence of a functional PI3K complex, Atg8 can be expressed and conjugated to PE, although autophagy will not take place (Itakura et al., 2008; Fogel et al., 2013). *VPS34*, a core subunit of PI3K, has been confirmed to be involved in the formation of autophagosomes in *C. neoformans* (Hu et al., 2008). Atg3 (E2 enzyme) and Atg4 (novel cysteine protease) were not important for the autophagy process in *C. neoformans*.

AMP-activated kinases (AMPKs) activate autophagy, whereas protein kinase A (PKA) and TORC1 inhibit autophagy (Stephan and Herman, 2006). During glucose starvation, AMPK is activated, whereas PKA is inhibited (Thevelein and Winde, 1999; Kamada et al., 2000; Aoh et al., 2013). Autophagy is induced in *S. cerevisiae* in response to abrupt carbon starvation, when cells are grown in the presence of glycerol, but not glucose, as the carbon source (Adachi et al., 2017). Consistent with these effects, autophagy was also induced under carbon starvation in *C. neoformans* (Figure 3B). The cleavage of GFP from the GFP-Atg8 fusion protein was hardly detected in *atg*7Δ, *atg*5Δ and *atg*12Δ, which are components of the ATG conjugation system and required for GFP cleavage, suggesting that lipidation of Atg8 is essential for its delivery to the vacuole. However, the cleavage of GFP from GFP-Atg8 was significantly enhanced compared to SD-N medium in other defective *ATG* strains. Carbon starvation also inactivates TORC1, which induced microautophagy resembling diauxic shift (carbon starvation)-induced microautophagy (Iwama and Ohsumi, 2019). The molecular mechanism of microautophagy does not require core Atg proteins but is regulated by the TORC1-Nem1/Spo7-Pah1 axis in budding yeast (Rahman et al., 2018). Therefore, we propose that the processing of GFP-Atg8 after long-term glucose starvation might be caused by microautophagy.

## Conclusion

In this study, we investigated the contribution of traditional core *ATG* genes to cryptococcal virulent factors, stress response, starvation tolerance, pathogenesis and autophagic flux in *C. neoformans*. The *atg*Δ strains displayed higher sensitivity to 1.5 M NaCl, lower thermotolerance and remarkably lower pathogenesis. Although autophagic flux depends on a few core complexes, it also displayed specific features under nitrogen and glucose starvation conditions (Supplementary Table S3). Overall, this study provides very useful information on the functions of *ATG* genes in *C. neoformans*.

## Data Availability Statement

The raw data supporting the conclusions of this manuscript will be made available by the authors, without undue reservation, to any qualified researcher.

## Author Contributions

XDZ, DW, and XZ conceived and designed the study. XZ, WF, XYZ, and CL performed the experiments. XM and XL provided assistance during in the experiments. XZ analyzed the data and wrote the manuscript. DW and XDZ reviewed and edited the manuscript. All authors read and approved the manuscript.

## Conflict of Interest

The authors declare that the research was conducted in the absence of any commercial or financial relationships that could be construed as a potential conflict of interest.

## Footnotes

1 https://mycocosm.jgi.doe.gov/Cryne_H99_1/Cryne_H99_1.home.html
