# Supplementary material for: Conserved Autophagy Pathway Contributes to Stress Tolerance and Virulence and Differentially Controls Autophagic Flux Upon Nutrient Starvation in Cryptococcus neoformans
Source: Front Microbiol. 2019 Nov 26;10:2690. doi: 10.3389/fmicb.2019.02690 (PMC6988817; doi:10.3389/fmicb.2019.02690)
Supplement: Supplementary file 1 [file Data_Sheet_1.docx]

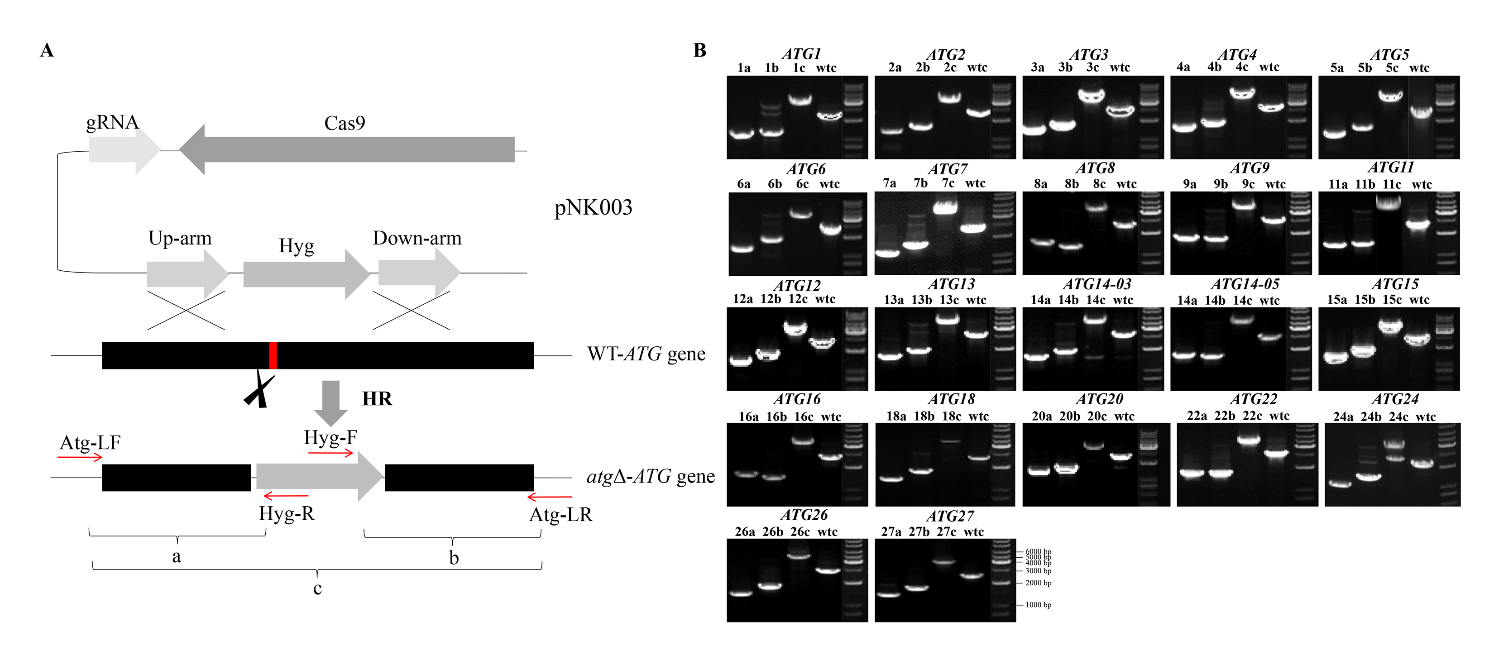


**Supplementary Figure 1.** Deletion and PCR verification of the *ATG* genes in *C. neoformans* **(A)** All targeted disruption mutants for *ATG* genes via a ‘suicide’ CRISPR-Cas9 system, the targets gRNA of *ATG* genes were indicated by a red rectangle, where a double-strand break was generated by Cas9 to promote homologous reorganization via double cross-over between *ATG* homologous arms, the primers used for PCR verification are indicated by the arrows. **(B)** Polymerase chain reaction (PCR) verification of the deletion in the mutants. Each gel photo represents one *ATG* gene. The primers Atg-LF/Hyg-R, Hyg-F/Atg-LR, and Atg-LF/ Atg-LR were used for the first lane, the second lane, the third and fourth lanes, respectively. The first three lanes are defective strains, and the fourth lane is wild type.

**
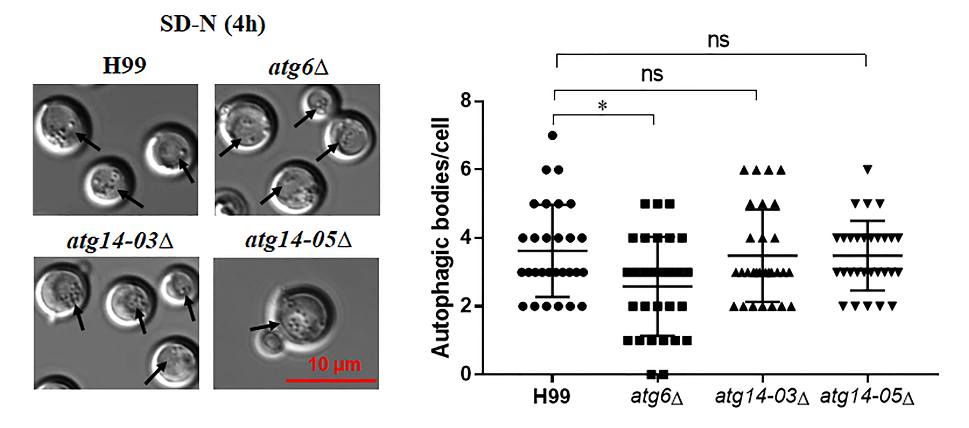
**

**Supplementary Figure 2.** Observation and quantification of autophagic body. **(A)** H99 and *atg*∆ strains were incubated in SD-N medium containing 1 mM PMSF and 10 μg/mL nocodazole for 4 h were harvested and subjected to DIC microscopy. Arrows point to autophagic bodies within vacuoles. **(B)** Quantification of autophagic bodies. The asterisk indicates the statistical significance of the comparison (*, *P* < 0.05, *t* test).

**Supplementary Figure 3.** Measurement of capsule sizes**.** H99 and *atg*∆ strains were incubated on maltose medium at 30°C for 4 days, cells were stained with India ink and measured with a Nikon Eclipse 80i fluorescence microscope (Nikon, Tokyo, Japan). The asterisk indicates the statistical significance of the comparison (*, *P* < 0.05, *t* test).


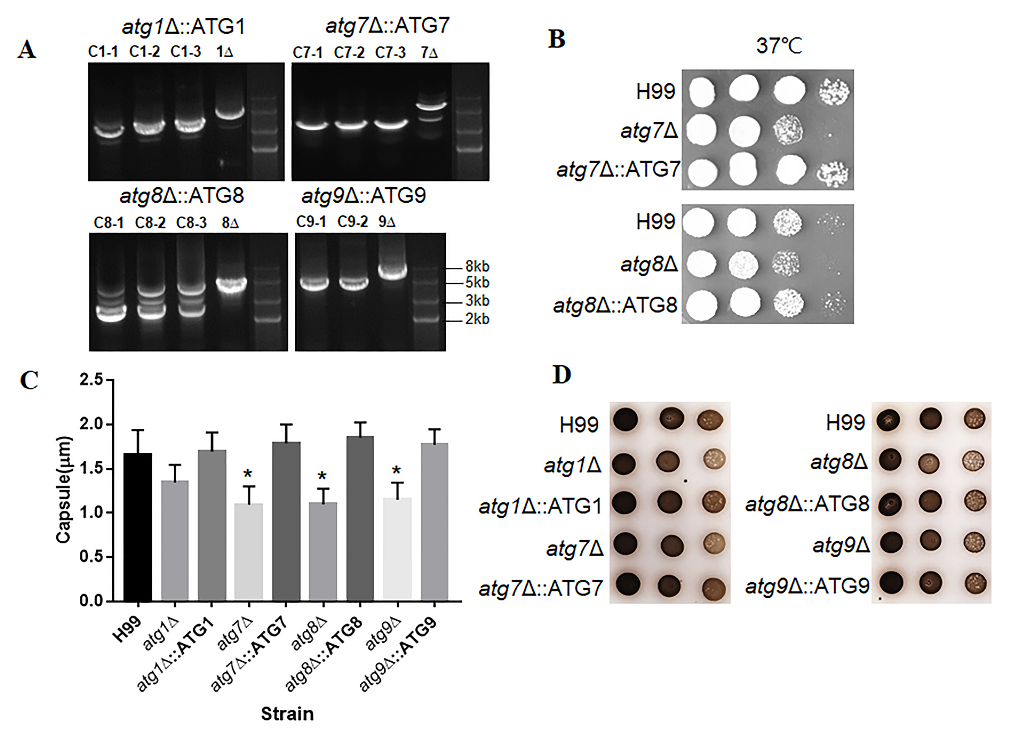


**Supplementary Figure 4.** Confirm the virulence factors of the *atg1*∆, *atg7*∆, *atg8*∆, and *atg9*∆ mutants. **(A)** PCR verification of the complementation strains. Each gel photo represents one *ATG* gene. The front lanes are the complement transformants, and the last one is the corresponding defective strain. (B) Thermotolerance test. Serial 10-fold dilutions of H99, *atg*∆ and complementation strains were spotted onto YPD medium and incubated at 37℃ for 3 days. **(C)** Capsule induction in low-iron medium at 30°C for 2 days. Cells were stained with India ink and measured with a Nikon Eclipse 80i fluorescence microscope (Nikon, Tokyo, Japan). The asterisk indicates the statistical significance of the comparison (*, *P* < 0.05, *t* test). **(D)** Melanin production on L-DOPA agar plates at 30°C for 3 days.

**
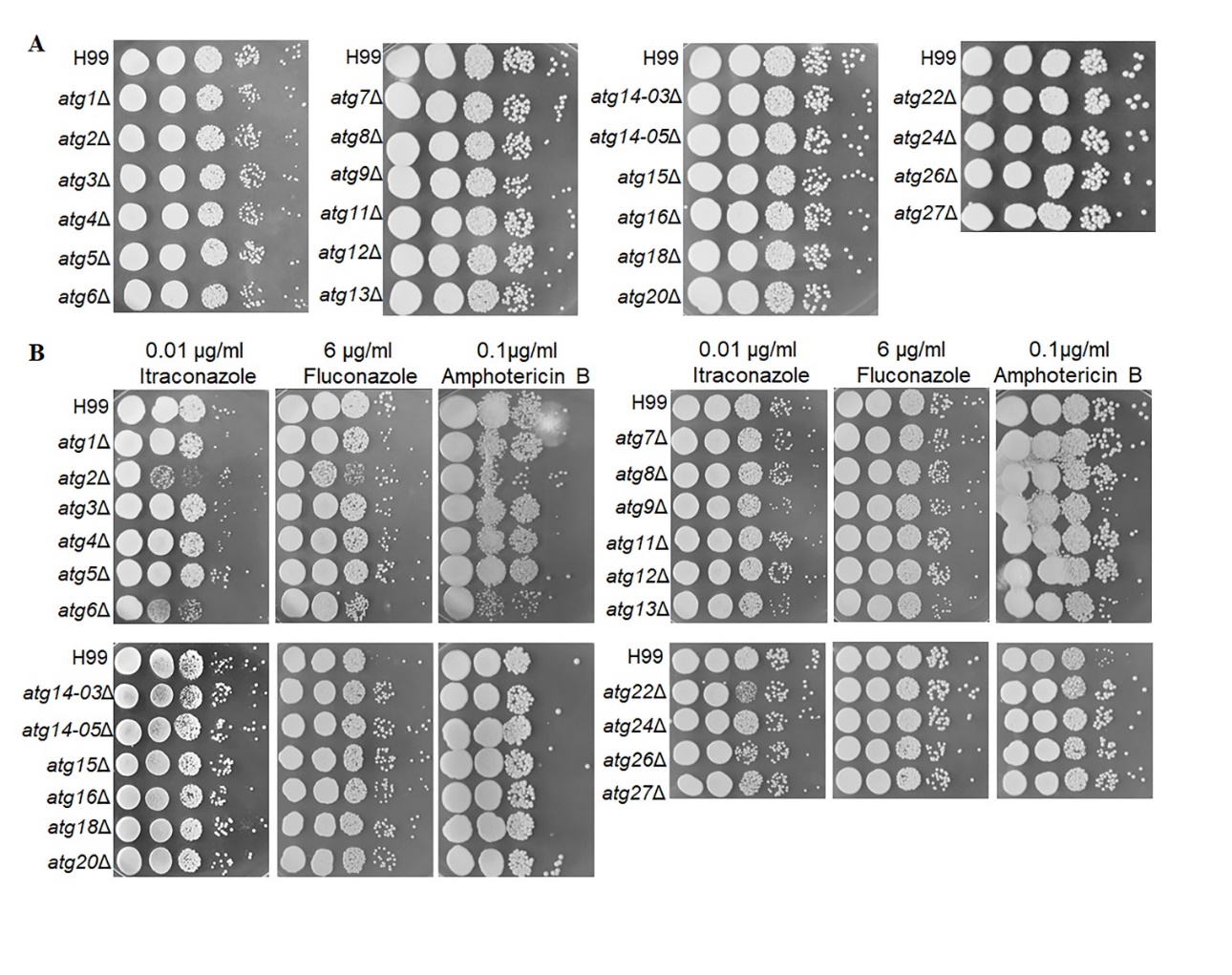
**

**Supplementary Figure 5.** *ATG* genes in antifungal resistance. Serial 10-fold dilutions of H99 and *atg*∆ strains were spotted on YPD medium **(A)** or containing 0.01 μg/mL itraconazole, 6 μg/mL fluconazole or 0.1μg/mL amphotericin B **(B)**, and incubated at 30°C for 3 days.
